# Sufficient Sample Size and Power in Multilevel Ordinal Logistic Regression Models

**DOI:** 10.1155/2016/7329158

**Published:** 2016-09-22

**Authors:** Sabz Ali, Amjad Ali, Sajjad Ahmad Khan, Sundas Hussain

**Affiliations:** ^1^Department of Statistics, Islamia College University, Peshawar, Pakistan; ^2^Department of Statistics, Abdul Wali Khan University Mardan, Khyber Pakhtunkhwa, Pakistan; ^3^Department of Statistics, Shaheed Benazir Bhutto Women University, Peshawar, Pakistan

## Abstract

For most of the time, biomedical researchers have been dealing with ordinal outcome variable in multilevel models where patients are nested in doctors. We can justifiably apply multilevel cumulative logit model, where the outcome variable represents the mild, severe, and extremely severe intensity of diseases like malaria and typhoid in the form of ordered categories. Based on our simulation conditions, Maximum Likelihood (ML) method is better than Penalized Quasilikelihood (PQL) method in three-category ordinal outcome variable. PQL method, however, performs equally well as ML method where five-category ordinal outcome variable is used. Further, to achieve power more than 0.80, at least 50 groups are required for both ML and PQL methods of estimation. It may be pointed out that, for five-category ordinal response variable model, the power of PQL method is slightly higher than the power of ML method.

## 1. Introduction

Data collected from hospitals and educational institutions are mostly multilevel or hierarchical data. This type of data is frequently used by researchers to construct statistical models such as multilevel models, hierarchical models, or mixed effects models [1, 2]. As the observations in these nested data structures become dependent, the classical methods and models like analysis of variance (ANOVA) and linear regression cannot be applied because these models assume independence. Hence, the use of alternative multilevel models is warranted to analyze the nested data structure.

It is really challenging to decide about an appropriate sample size for multilevel ordinal logistic models. In the contemporary literature, only [3] discusses the issue of sample size in multilevel ordinal logistic model by using PQL method of estimation. The researcher uses three-category multilevel ordinal logistic models. Apart from this, there is no existing research on sample size and power issues in multilevel ordinal logistic models. Unlike [3], the study of [4] compares both PQL and ML methods in small group sizes. However, the study of [4] does not provide any results about power analysis. In the present study, the focus of researchers is not only to compare ML and PQL estimation methods of estimation in larger group sizes but also to provide guidelines about optimum sample size needed for multilevel ordinal logistic models.

## 2. Materials and Methods

### 2.1. Multilevel Logistic Regression Model

A very popular concept is used in social sciences to develop a dichotomous multilevel logistic model through a latent continuous variable model [5]. The same idea can be extended to three or more ordered categories through a threshold parameters. A threshold concept is used that the latent continuous variable *Y*_*ij*_^*∗*^ underlies the observed variable *Y*_*ij*_. A simple two level ordinal logistic model can be written as

(1) Yij∗=β0j+β1jXij+eijlevel  1  modelβ0j=γ00+γ01Wj+uojlevel  2  modelβ1j=γ10+γ11Wj+u1jYij∗=γ00+γ10Xij+γ01Wj+γ11XijWj+uoj+u1jXij+eijFixed  part+Random  part,

where *X*_*ij*_ corresponds to level 1 explanatory variable, *W*_*j*_ represents level 2 explanatory variable, and level 1 coefficients denoted by *β* and *γ*'s are the fixed effects. If it is assumed that *e*_*ij*_ follows a normal distribution, that is, *e*_*ij*_ ~ *N*(0,1), then the resulting multilevel model is termed as multilevel probit model. Similarly, if *e*_*ij*_ ~ logistic(0, *π* ^2^/3), then the model is said to be multilevel logistic model [6]. Level 2 random effects are more often assumed to have a normal distribution as

(2) $$\begin{array}{c} \left[\begin{array}{c} u_{oj}\\ u_{1j} \end{array}\right]\sim N\left(\;\left[\begin{array}{c} 0\\ 0 \end{array}\right],\left[\begin{array}{cc} \sigma_{u}^{2} & \sigma_{u1}\\ \sigma_{u1} & \sigma_{1}^{2} \end{array}\right]\;\right). \end{array}$$

According to [7], ICC is the proportion of group level variance compared to the total variance, represented by

(3) $$\begin{array}{c} \text{ICC}=\frac{\sigma_{u}^{2}}{\sigma_{u}^{2}+\pi^{2}/3}. \end{array}$$

*σ*_*u*_^2^ is the group level or level 2 variance and *π* ^2^/3 is the individual level or level 1 variance so the total variance = (level 2 variance) + (level 1 variance) = *σ*_*u*_^2^ + *π* ^2^/3.

That is why the squared sigma *u*(*σ*_*u*_^2^) appeared in both numerator and denominator.

Now *Y*_*ij*_^*∗*^ can be linked with the observed variable *Y*_*ij*_ through a threshold model. The threshold model for *m* categories, *m* = 1,2, 3,…, *M*, can be written as

(4) $$\begin{array}{c} Y_{ij}=1\;\text{if }Y_{ij}^{*}<v^{\left(1\right)}\\ Y_{ij}=2\;\text{if }v^{\left(1\right)}\leq Y_{ij}^{*}\leq v^{\left(2\right)}\\ Y_{ij}=3\;\text{if }v^{\left(2\right)}\leq Y_{ij}^{*}\leq v^{\left(3\right)}\\ \vdots \\ Y_{ij}=M\;\text{if }Y_{ij}^{*}\geq v^{\left(M-1\right)}, \end{array}$$

where (*v* ^(1)^ < *v* ^(2)^ < *v* ^(3)^ ⋯ <*v* ^(*M*−1)^) are the threshold parameters.

For identification purpose, it is common to set the first threshold to zero and to allow an intercept in the model [8]. We will assume a proportional odds model which means that the effect of explanatory variables will remain the same across categories [9–12]. The equivalent to the cumulative logit model above in generalized linear models context is

(5) $$\begin{array}{c} \eta_{ij}=\gamma_{00}+\gamma_{10}X_{ij}+\gamma_{01}W_{j}+\gamma_{11}X_{ij}W_{j}+u_{oj}+u_{1j}X_{ij}. \end{array}$$

### 2.2. Simulation Design

The fixed effect parameters (*γ*_00_, *γ*_10_, *γ*_01_, *γ*_11_) were set from the previous study by [4]. *γ*_10_ = 1, *γ*_01_ = 1, *γ*_11_ = 1, and *γ*_00_ = 0. The reason behind *γ*_00_ = 0 is that multilevel ordinal logistic model cannot identify the overall model intercept and all threshold parameters jointly. There are two ways: a researcher can (1) estimate the intercept by setting the first threshold parameter to zero or (2) equate the intercept to zero and estimate the threshold parameters. That is why we put *γ*_00_ = 0. There are three groups conditions (30,50,100), three group sizes (5,30,50), and three values of ICC (0.1,0.3,0.5). These values of ICC correspond to an intercept variance *σ*_*u*_^2^ = 0.39,1.45, and 3.4. Similarly, random slope values were taken as *σ*_1_^2^ = 0.12,0.32, and 0.62, while *σ*_*u*1_^2^ = 0.01,0.15, and 0.30. For each scenario, the number of simulation R was set to be 1000. We used GLIMMIX (ML) with adaptive quadrature procedure and GLLIMIX (PQL) procedures in SAS for data generation and analyses.

### 2.3. Procedure for the Parameter Estimations

The accuracy of different fixed effect and random effect parameters estimates was calculated through the relative parameter bias, that is,

(6) $$\begin{array}{c} RPB=\frac{Estimate-parameter}{Parameter}, \end{array}$$

while estimate is the value produced by ML or PQL method of estimations and parameter values are those taken in the simulation design. Average relative biases for Tables 1 and 2 are obtained by using (6).

Similarly, power was computed as

(7) $$\begin{array}{c} \frac{Estimate}{Standard\;\;error}. \end{array}$$

Empirical powers were computed for *X*_*ij*_, *W*_*j*_, and *X*_*ij*_ *W*_*j*_ by using (7). The power was calculated as the number of replications in which *H*_0_ : Parameter  value = 0, was correctly rejected at 5 percent level of significance divided by 1000 as we used 1000 replications for each condition. Power values between 0.80 and 1.0 were considered excellent and below 0.80 as inadequate [13]. For brevity, the threshold estimates are excluded as researchers are not interested in their values.

Empirical coverage rates of 95% confidence intervals were used to judge the accuracy of the standard errors of estimated parameters [14]. Tables 4 [Table tab5] [Table tab6] [Table tab7] [Table tab8] [Table tab9] [Table tab10]–11 were obtained by using

(8) $$\begin{array}{c} Estimate\pm Z_{\alpha /2}\times \text{standard error of estimate}. \end{array}$$

The 95% confidence intervals coverage rates were computed in each condition as the proportion of replications in which the true parameter is captured by the 95% confidence interval. Reference [15] recommended acceptable coverage rates as 92.5% to 97.5%. A separate logistic regression was used to assess the impact of simulation conditions on empirical coverage rates of estimates. *P* values in Tables 4 to 11 are obtained by

(9) $$\begin{array}{c} \text{logit}\left(\;P_{ij}\;\right)=\beta_{0}+\beta_{1}\text{groups}+\beta_{2}\text{group size}+\beta_{3}\text{ICC}. \end{array}$$

## 3. Results and Discussion

Tables 1 and 2 are estimated for the purpose of checking the accuracy of both fixed and random effects estimates through relative bias. Absolute relative bias <5% is negligible under both ML and PQL method. Estimates that have absolute relative bias close to zero are considered as unbiased estimates; that is, estimates and parameters become identical.

Tables 4 to 11 are computed to check the accuracy of estimates standard errors through 95% confidence interval. Those estimates which achieve acceptable coverage rates (92.5% to 97.5%) are considered best. Similarly, Table 3 is computed to get power rates under both ML and PQL methods.

Table 1 represents the impact of various simulation conditions on average relative bias of the fixed effect estimates under two estimation methods ML and PQL. Estimates were substantially biased when the number of groups was 30, group size was 5, and ICC = 0.1 under ML method. The estimates relative bias was less than 5% when the number of groups was 50 under ML method. With 100 groups, estimates were unbiased under ML method. Estimates relative bias was negligible when the number of groups was 100 under ML method. Relative bias of estimates was highly influenced by the number of groups under ML method. On the contrary, estimates were underestimated under PQL method. Substantial bias of estimates was noted when group size was 5 and ICC = 0.5 under PQL method. Estimates relative bias was less than 5% when group size was 50 under PQL method. On average, ML method fixed effects estimates average relative bias was smaller than that of PQL method in absolute terms. Moreover, group size impact was larger on estimates relative bias under PQL method.

Similarly, ML method random effects estimates were substantially biased when the number of groups was 30 as shown in Table 3. With 100 groups, random effects estimates bias was less than 5% consistently. ML method performed well when the number of groups was large. On the other hand, random effects estimates were substantially biased under PQL method when group size was 5 and ICC = 0.5. Like fixed effects estimates, ML method random effects bias was smaller than that of PQL method random effects estimates bias in absolute terms.

Table 3 reflects the power pattern of estimates under both ML and PQL methods in five-category ordinal outcome variable model. Further, to achieve more than 0.80 power rates for estimates, at least 50 groups are mandatory for both estimation methods. However, the power of PQL method estimates was slightly higher than that of ML method estimates in five-category ordinal outcome variable model.

Table 4 lists empirical coverage rates for estimates of the multilevel ordinal logistic model under ML method when response variable had three categories and distribution type was symmetrical. Under the three group conditions, the fixed effect estimates achieve the acceptable coverage rates (92.5 to 97.5) defined by [13]. However, random effect estimates coverage rates were smaller than the nominal coverage rates. The effect of the number of groups was significant on estimates coverage rates. However, group size and ICC effect was minimal on estimates coverage rates. Table 4 results suggest that a large number of groups should be used rather than larger group size under ML method.

Table 5 lists empirical coverage rates for estimates of the multilevel ordinal logistic model under ML method when response variable had three categories and distribution type was skewed. Again, group size and ICC effect was minimal on estimates empirical coverage rates. The impact of the number of groups was again significant and dominant on estimates empirical coverage rates. Moreover, to achieve unbiased standard errors of estimates, large number of groups will be better than the larger group size under ML method.

Table 6 lists empirical coverage rates for estimates of the multilevel ordinal logistic model under ML method when response variable had five categories and distribution type was symmetrical. Like three-category response model, the influence of the number of groups was significant on estimates coverage rates under ML method. More groups should be used to achieve the desired results under ML method.

Table 7 lists empirical coverage rates for estimates of the multilevel ordinal logistic model under ML method when response variable had five categories and distribution type was skewed. Like five-category response model, the influence of the number of groups was again significant on estimates coverage rates under ML method. On the other hand, group size and ICC effect was insignificant on estimates empirical coverage rates under ML method.

Table 8 lists empirical coverage rates for estimates of the multilevel ordinal logistic model under PQL method when response variable had three categories and distribution type was symmetrical. With group size 5, fixed effects estimates coverage rates were unacceptable. However, with group size of 30 and 50, respectively, fixed estimates coverage rates were acceptable. Furthermore, random effects estimates coverage rates were unacceptable across all conditions. Additionally, ICC had also a significant effect on estimates coverage rates; that is, coverage rates decreased with the larger ICC values under PQL method. On the other hand, groups effect was little under PQL method on estimates coverage rates.

Table 9 lists empirical coverage rates for estimates of the multilevel ordinal logistic model under PQL method when response variable had three categories and distribution type was skewed. Group size factor had a significant effect on estimates coverage rates. However, coverage rates consistently decreased with the increase in ICC values under PQL method. Fixed effects achieved acceptable coverage rates across the last two levels of group size factor, that is, 30 and 50, respectively. Groups had a significant effect on coverage rates. Larger group sizes seem to be good under PQL method.

Table 10 lists empirical coverage rates for estimates of the multilevel ordinal logistic model under PQL method when response variable had five categories and distribution type was symmetrical. Fixed effects achieved acceptable coverage rates across all group sizes, definitely reflecting improved performance of PQL method over three-category response model. The performance of PQL method was as good as ML method in five-category response model. However, estimates coverage rates decreased with the larger values of ICC. Again, the effect of the number of groups was insignificant on coverage rates.

Table 11 lists empirical coverage rates for estimates of the multilevel ordinal logistic model under PQL method when response variable had five categories and distribution type was skewed. Fixed effect coverage rates were all acceptable across all group sizes. However, random effects coverage was unacceptable and smaller than that of ML coverage rates. Group size again showed a significant effects on all estimates coverage rates while groups effect was insignificant.

## 4. Conclusion

In both symmetrical and skewed distribution shapes of category responses, *γ*_10_, *γ*_01_, and *γ*_11_ were overestimated in majority of the conditions under ML method of estimation when three-category and five-category multilevel ordinal logistic models were used. Moreover, random effects estimates, that is, random intercept and random slope estimates, were more biased than fixed effects estimates on average. The random effects estimates were not substantially biased under ML method of estimation, especially when the number of groups was large and random effects were not small. On the contrary, *γ*_10_, *γ*_01_, and *γ*_11_ were underestimated almost across all conditions under PQL method of estimation when three-category and five-category multilevel ordinal logistic models were used. Like ML method of estimation, random effects estimates relative biases were higher on average than those of the fixed effects estimates under PQL method. Both fixed effects and random effects estimates were not substantially biased under PQL method of estimation, particularly when population random effects were small (*σ*_*u*_^2^ = 0.39, *σ*_1_^2^ = 0.12, and *σ*_*u*1_ = 0.01) and group sizes were large. In general, the absolute relative bias of ML method estimates was consistently smaller than that of PQL method estimates.

The accuracy of standard errors of the estimates (excluding threshold estimates) was judged through empirical coverage rates. The influence of the number of groups was significant on the accuracy of both three-category and five-category multilevel ordinal logistic models estimates standard errors under ML method of estimation. On the contrary, the group size factor and ICC had an insignificant effect on estimates standard errors under ML method of estimation. Estimates standard errors were least biased when number of groups was 100. On the other hand, the influence of group size factor was highly significant on the accuracy of estimates standard errors under PQL method of estimation. Furthermore, ICC also influenced estimates standard errors; that is, standard errors were substantially biased when population random effects were medium (*σ*_*u*_^2^ = 1.45, *σ*_1_^2^ = 0.32, and *σ*_*u*1_ = 0.15) and large (*σ*_*u*_^2^ = 3.4, *σ*_1_^2^ = 0.62, and *σ*_*u*1_ = 0.30). The impact of the number of groups on estimates standard errors in majority of the conditions was minimal under PQL method. However, five-category multilevel ordinal logistic model estimates standard errors were as good as that of ML method.

The power rates of PQL estimates were slightly higher than that of ML estimates when ordinal response variable had five categories, which indicate that PQL standard errors were least biased due to increases in the number of categories of ordinal response variable.

In general, ML method performed well in terms of estimates small bias, high coverage rates, and high power rates when ordinal response variable had three categories. However, in five-category ordinal response variable model, PQL method performances were comparable to those of ML method. PQL estimates were poor when population random effects (ICC) were medium and large while ML estimates were poor in small population random effects. In addition, ML estimates and estimates standard errors benefitted from larger number of groups while PQL estimates and estimates standard errors benefitted from larger group sizes. We recommend at least 100 groups and 30 units per group to achieve accurate multilevel ordinal logistic model estimates and estimates standard errors when method of estimation is ML. Furthermore, 100 groups and at least 50 units per group are essential for accurate multilevel ordinal logistic model estimates and estimates standard errors when method of estimation is PQL. Similarly, at least 50 groups are essential to achieve 0.80 or more power for both ML and PQL methods of estimation. On the basis of this study, it is recommended that PQL method may be avoided when group sizes are small, number of groups are large, random effects are medium and large, and outcome variable has three categories. In such conditions, ML method is the best option. However, when the outcome variable has five or more categories, random effects are small, group sizes are large, and number of groups is small, PQL method may be better option.

## Figures and Tables

**Table 1:** Average relative bias of fixed effect estimates obtained as a function of groups, group size, and ICC, collapsing over the category distribution and number of categories.

Groups	Group size	ICC	ML method			PQL method		
			*γ*_10_	*γ*_01_	*γ*_11_	*γ*_10_	*γ*_01_	*γ*_11_
30	5	0.1	0.0953	0.0960	0.0523	−0.0925	−0.0858	−0.0810
		0.3	0.0790	0.0823	0.0397	−0.1061	−0.0967	−0.0899
		0.5	0.0636	0.0675	0.0313	−0.1186	−0.1074	−0.0999

30	30	0.1	0.0708	0.0376	0.0469	−0.0601	−0.0595	−0.0603
		0.3	0.0577	0.0292	0.0496	−0.0677	−0.0714	−0.0797
		0.5	0.0506	0.0263	0.0347	−0.0732	−0.0816	−0.0878

30	50	0.1	0.0314	0.0562	0.0785	−0.0427	−0.0433	−0.0452
		0.3	0.0256	0.0507	0.0778	−0.0539	−0.0551	−0.0720
		0.5	0.0161	0.0466	0.0696	−0.0642	−0.0598	−0.0829

50	5	0.1	0.0750	0.0607	0.0656	−0.0587	−0.0616	−0.0678
		0.3	0.0577	0.0485	0.0558	−0.0645	−0.0669	−0.0763
		0.5	0.0471	0.0489	0.0423	−0.0742	−0.0690	−0.0796

50	30	0.1	0.0175	0.0196	0.0285	−0.0410	−0.0518	−0.0506
		0.3	0.0202	0.0154	0.0219	−0.0474	−0.0443	−0.0559
		0.5	0.0160	0.0154	0.0337	−0.0536	−0.0570	−0.0611

50	50	0.1	0.0281	0.0248	0.0501	−0.0252	−0.0384	−0.0400
		0.3	0.0340	0.0260	0.0333	−0.0391	−0.0370	−0.0447
		0.5	0.0265	0.0243	0.0307	−0.0412	−0.0402	−0.0400

100	5	0.1	0.0319	0.0357	0.0421	−0.0520	−0.0505	−0.0472
		0.3	0.0188	0.0169	0.0255	−0.0549	−0.0416	−0.0357
		0.5	0.0235	0.0188	0.0211	−0.0518	−0.0536	−0.0303

100	30	0.1	−0.0025	0.0066	0.0092	−0.0206	−0.0270	−0.0236
		0.3	−0.0033	0.0044	0.0060	−0.0216	−0.0324	−0.0230
		0.5	−0.0037	0.0019	0.0041	−0.0248	−0.0172	−0.0182

100	50	0.1	0.0064	0.0069	0.0041	−0.0102	−0.0114	−0.0084
		0.3	0.0051	0.0048	0.0025	−0.0105	−0.0125	−0.0243
		0.5	0.0030	0.0031	0.0042	−0.0148	−0.0120	−0.0205

**Table 2:** Average relative bias of random effect estimates obtained as a function of groups, group size, and ICC, collapsed over the category distribution and number of categories.

Groups	Group size	ICC	ML method		PQL method	
			*σ*_*u*_	*σ*_1_	*σ*_*u*_	*σ*_1_
30	5	0.1	−0.1106	0.2000	−0.0812	0.1143
		0.3	−0.0701	−0.0176	−0.1158	−0.1403
		0.5	−0.0326	0 .0127	−0.1469	−0.1725

30	30	0.1	−0.0806	−0.0572	−0.0645	−0.1429
		0.3	−0.0578	−0.0702	−0.0926	−0.1053
		0.5	−0.0327	−0.0633	−0.1196	−0.1311

30	50	0.1	−0.0645	−0.0858	−0.0483	−0.1066
		0.3	−0.0578	−0.0351	−0.0689	−0.0878
		0.5	−0.0490	−0.0620	−0.0794	−0.1079

50	5	0.1	−0.0645	0.0286	−0.0967	0.0282
		0.3	−0.0330	−0.0702	−0.1239	−0.1555
		0.5	0.0226	−0.0507	−0.1414	−0.1809

50	30	0.1	−0.0483	−0.1143	−0.0725	−0.1525
		0.3	−0.0413	−0.0527	−0.0893	−0.1209
		0.5	−0.0272	−0.0380	−0.1022	−0.1461

50	50	0.1	−0.0483	−0.0572	−0.0512	−0.0882
		0.3	−0.0330	−0.0176	−0.0656	−0.0933
		0.5	−0.0272	−0.0291	−0.0751	−0.1131

100	5	0.1	−0.0322	0.0000	−0.0754	−0.1220
		0.3	−0.0330	−0.0179	−0.1063	−0.1425
		0.5	0.0055	−0.0254	−0.1095	−0.1520

100	30	0.1	−0.0322	−0.0286	−0.0442	−0.0832
		0.3	−0.0328	−0.0176	−0.0611	−0.1051
		0.5	−0.0055	0.0000	−0.0709	−0.1165

100	50	0.1	−0.0330	0.0000	−0.0391	−0.0595
		0.3	−0.0333	−0.0180	−0.0476	−0.0682
		0.5	−0.0540	0.0000	−0.0465	−0.0729

**Table 3:** Power of fixed effect estimates of five-category response multilevel ordinal logistic model (method: ML and PQL, category distribution: symmetrical).

Groups	Group size	ICC	ML method			PQL method		
			*γ*_10_	*γ*_01_	*γ*_11_	*γ*_10_	*γ*_01_	*γ*_11_
30	5	0.1	0.536	0.551	0.488	0.607	0.611	0.523
		0.3	0.561	0.559	0.492	0.596	0.621	0.539
		0.5	0.572	0.550	0.501	0.599	0.616	0.531

30	30	0.1	0.624	0.661	0.569	0.661	0.659	0.591
		0.3	0.639	0.639	0.589	0.650	0.672	0.602
		0.5	0.635	0.669	0.594	0.658	0.649	0.611

30	50	0.1	0.715	0.709	0.661	0.739	0.693	0.681
		0.3	0.731	0.721	0.679	0.749	0.716	0.699
		0.5	0.739	0.732	0.703	0.745	0.726	0.693

50	5	0.1	0.850	0.835	0.731	0.886	0.849	0.756
		0.3	0.861	0.849	0.729	0.897	0.863	0.769
		0.5	0.873	0.858	0.740	0.897	0.869	0.763

50	30	0.1	0.871	0.864	0.778	0.903	0.914	0.809
		0.3	0.877	0.856	0.789	0.914	0.895	0.795
		0.5	0.869	0.879	0.768	0.909	0.899	0.799

50	50	0.1	0.891	0.859	0.815	0.956	0.916	0.849
		0.3	0.882	0.880	0.826	0.969	0.931	0.871
		0.5	0.896	0.878	0.831	0.972	0.939	0.878

100	5	0.1	0.992	1.000	0.851	1.000	1.000	0.914
		0.3	0.998	1.000	0.859	1.000	0.997	0.902
		0.5	0.998	0.995	0.867	1.000	1.000	0.909

100	30	0.1	0.995	0.989	0.890	1.000	1.000	0.929
		0.3	0.997	0.998	0.897	1.000	1.000	0.936
		0.5	0.992	0.998	0.879	1.000	1.000	0.939

100	50	0.1	0.989	0.992	0.906	1.000	1.000	0.952
		0.3	0.996	0.996	0.919	1.000	1.000	0.959
		0.5	0.996	0.997	0.899	1.000	1.000	0.951

**Table 4:** 95% CI coverage rates for the estimates in a three-category ordinal response variable model by groups, group size, and intraclass correlation (method: ML, category distribution: symmetrical).

Parameters	Number of groups				Group size				ICC			
	30	100	120	*P* value	5	30	50	*P* value	0.1	0.3	0.5	*P* value
*γ*_10_	0.942	0.949	0.953	0.0006	0.948	0.946	0.949	0.7886	0.945	0.948	0.951	0.0270
*γ*_01_	0.941	0.947	0.952	0.0008	0.946	0.946	0.950	0.3865	0.943	0.947	0.951	0.0165
*γ*_11_	0.939	0.946	0.951	0.0007	0.945	0.945	0.947	0.4895	0.946	0.946	0.946	0.9737
*σ*_*u*_	0.905	0.908	0.918	0.0041	0.908	0.910	0.913	0.3086	0.911	0.910	0.909	0.6199
*σ*_1_	0.907	0.915	0.927	0.0000	0.912	0.917	0.920	0.0632	0.918	0.913	0.918	0.8929

**Table 5 tab5:** 95% CI coverage rates for the estimates in a three-category ordinal response variable model by groups, group size, and intraclass correlation (method: ML, category distribution: skewed).

Parameters	Number of groups				Group size				ICC			
	30	100	120	*P* value	5	30	50	*P* value	0.1	0.3	0.5	*P* value
*γ*_10_	0.941	0.948	0.951	0.0053	0.946	0.948	0.949	0.5283	0.943	0.948	0.949	0.0503
*γ*_01_	0.946	0.949	0.951	0.1780	0.947	0.948	0.950	0.4000	0.947	0.948	0.949	0.7360
*γ*_11_	0.942	0.946	0.955	0.0001	0.944	0.950	0.949	0.0821	0.948	0.947	0.948	0.9733
*σ*_*u*_	0.900	0.908	0.917	0.0000	0.904	0.908	0.913	0.0494	0.908	0.908	0.910	0.7760
*σ*_1_	0.909	0.913	0.922	0.0011	0.909	0.915	0.920	0.0077	0.918	0.913	0.918	0.8939

**Table 6 tab6:** 95% CI coverage rates for the estimates in a five-category ordinal response variable model by groups, group size, and intraclass correlation (method: ML, category distribution: symmetrical).

Parameters	Number of groups				Group size				ICC			
	30	100	120	*P* value	5	30	50	*P* value	0.1	0.3	0.5	*P* value
*γ*_10_	0.946	0.948	0.950	0.2542	0.945	0.949	0.951	0.0702	0.947	0.946	0.951	0.3144
*γ*_01_	0.945	0.946	0.949	0.4840	0.945	0.947	0.948	0.2440	0.947	0.946	0.947	0.9730
*γ*_11_	0.943	0.947	0.949	0.0490	0.946	0.947	0.946	0.9470	0.948	0.944	0.948	0.9210
*σ*_*u*_	0.904	0.907	0.918	0.0001	0.909	0.910	0.912	0.4497	0.907	0.912	0.912	0.2305
*σ*_1_	0.903	0.915	0.926	0.0000	0.911	0.914	0.918	0.0585	0.914	0.915	0.914	1.0000

**Table 7 tab7:** 95% CI coverage rates for the estimates in a five-category ordinal response variable model by groups, group size, and intraclass correlation (method: ML, category distribution: skewed).

Parameters	Number of groups				Group size				ICC			
	30	100	120	*P* value	5	30	50	*P* value	0.1	0.3	0.5	*P* value
*γ*_10_	0.939	0.944	0.946	0.0340	0.947	0.933	0.948	0.8470	0.944	0.943	0.941	0.3860
*γ*_01_	0.945	0.947	0.949	0.1940	0.951	0.937	0.953	0.6170	0.947	0.947	0.947	0.9470
*γ*_11_	0.944	0.947	0.948	0.1650	0.948	0.939	0.951	0.4080	0.944	0.947	0.947	0.3720
*σ*_*u*_	0.903	0.911	0.916	0.0024	0.910	0.904	0.917	0.0945	0.908	0.911	0.912	0.3885
*σ*_1_	0.907	0.916	0.922	0.0003	0.912	0.910	0.922	0.0328	0.913	0.915	0.916	0.5935

**Table 8 tab8:** 95% CI coverage rates for the estimates in a three-category ordinal response variable model by groups, group size, and intraclass correlation (method: PQL, category distribution: symmetrical).

Parameters	Number of groups				Group size				ICC			
	30	100	120	*P* value	5	30	50	*P* value	0.1	0.3	0.5	*P* value
*γ*_10_	0.921	0.925	0.928	0.1135	0.918	0.926	0.932	0.0030	0.925	0.926	0.922	0.5156
*γ*_01_	0.918	0.922	0.925	0.0671	0.917	0.922	0.928	0.0170	0.928	0.924	0.912	0.0001
*γ*_11_	0.923	0.927	0.929	0.1099	0.920	0.926	0.933	0.0014	0.931	0.929	0.918	0.0009
*σ*_*u*_	0.897	0.900	0.907	0.0087	0.896	0.902	0.908	0.0044	0.905	0.903	0.895	0.0215
*σ*_1_	0.903	0.906	0.908	0.1327	0.898	0.906	0.912	0.0018	0.910	0.909	0.897	0.0055

**Table 9 tab9:** 95% CI coverage rates for the estimates in a three-category ordinal response variable model by groups, group size, and intraclass correlation (method: PQL, category distribution: skewed).

Parameters	Number of groups				Group size				ICC			
	30	100	120	*P* value	5	30	50	*P* value	0.1	0.3	0.5	*P* value
*γ*_10_	0.918	0.920	0.924	0.1155	0.915	0.919	0.927	0.0053	0.928	0.923	0.912	0.0000
*γ*_01_	0.921	0.926	0.928	0.0245	0.920	0.927	0.931	0.0044	0.931	0.929	0.917	0.0002
*γ*_11_	0.921	0.924	0.928	0.0965	0.918	0.924	0.931	0.0031	0.929	0.926	0.918	0.0032
*σ*_*u*_	0.899	0.902	0.905	0.0567	0.897	0.902	0.907	0.0274	0.903	0.905	0.898	0.1459
*σ*_1_	0.905	0.908	0.911	0.1633	0.901	0.910	0.914	0.0015	0.916	0.909	0.900	0.0003

**Table 10 tab10:** 95% CI coverage rates for the estimates in a five-category ordinal response variable model by groups, group size, and intraclass correlation (method: PQL, category distribution: symmetrical).

Parameters	Number of groups				Group size				ICC			
	30	100	120	*P* value	5	30	50	*P* value	0.1	0.3	0.5	*P* value
*γ*_10_	0.940	0.945	0.942	0.6312	0.936	0.942	0.948	0.0007	0.944	0.945	0.937	0.0590
*γ*_01_	0.940	0.945	0.946	0.0997	0.938	0.940	0.948	0.0027	0.945	0.945	0.940	0.1292
*γ*_11_	0.938	0.944	0.942	0.2029	0.937	0.942	0.946	0.0156	0.943	0.942	0.939	0.2029
*σ*_*u*_	0.906	0.910	0.911	0.2328	0.904	0.910	0.914	0.0458	0.913	0.911	0.903	0.0196
*σ*_1_	0.907	0.907	0.909	0.6620	0.904	0.909	0.910	0.2170	0.909	0.910	0.902	0.1050

**Table 11:** 95% CI coverage rates for the estimates in a five-category ordinal response variable model by groups, group size, and intraclass correlation (method: PQL, category distribution: skewed).

Parameters	Number of groups				Group size				ICC			
	30	100	120	*P* value	5	30	50	*P* value	0.1	0.3	0.5	*P* value
*γ*_10_	0.941	0.941	0.943	0.5893	0.937	0.942	0.946	0.0047	0.944	0.943	0.937	0.0611
*γ*_01_	0.939	0.940	0.941	0.5301	0.935	0.940	0.946	0.0013	0.942	0.941	0.937	0.1578
*γ*_11_	0.942	0.943	0.944	0.6300	0.936	0.944	0.950	0.0000	0.946	0.944	0.938	0.0160
*σ*_*u*_	0.903	0.907	0.909	0.1598	0.901	0.908	0.910	0.0658	0.907	0.907	0.904	0.6272
*σ*_1_	0.909	0.911	0.914	0.3463	0.905	0.911	0.917	0.0060	0.915	0.915	0.903	0.0037
